# Case Report: Therapeutic Response to Chemo-Immunotherapy in an Advanced Large Cell Lung Carcinoma Patient With Low Values of Multiple Predictive Biomarkers

**DOI:** 10.3389/fimmu.2020.607416

**Published:** 2021-01-28

**Authors:** Guihua Wang, Qin Chai, Yajie Xiao, Wenying Peng, Miao Teng, Jingyi Wang, Hanqing Lin, Xiaofan Su, Lin Wu

**Affiliations:** ^1^ Department of Oncology, Changsha Central Hospital, Changsha, China; ^2^ YuceBio Technology Co., Ltd., Shenzhen, China; ^3^ The Second Department of Thoracic Oncology, Hunan Cancer Hospital, The Affiliated Cancer Hospital of Xiangya School of Medicine, Central South University, Changsha, China; ^4^ The Second Department of Oncology, Yunnan Cancer Hospital, Kunming, China

**Keywords:** large cell lung carcinoma, PD-L1 expression, tumor mutational burden, CD8(+) tumor-infiltrating lymphocytes, chemo-immunotherapy, predictive biomarker

## Abstract

Immune checkpoint inhibitors have revolutionized the treatments of lung cancers, and multiple predictive biomarkers alone or in combination help clinicians with the appropriate therapeutic selections. Recently, chemo-immunotherapy has been recommended for treating advanced non-small cell lung cancers in patients without driver mutations. However, the clinical relevance of predictive biomarkers and the treatment efficacy of chemo-immunotherapy in large cell lung carcinoma (LCLC) remain unclear. Here, we reported a rare case of LCLC with none driver gene mutations and low values of multiple predictive biomarkers. These biomarkers included a low PD-L1 expression of 5–10%, a low tumor mutational burden (TMB) of 2.5 muts/mb, a low CD8(+) tumor-infiltrating lymphocyte density of 147.91 psc/mm². After one-cycle chemotherapy, the patient progressed rapidly and then was switched to pembrolizumab combining paclitaxel plus cisplatin. Interestingly, he achieved a partial response after two cycles of chemo-immunotherapy, showing multiple lymph nodes obviously shrunk on CT scan, and other clinical symptoms were relieved when compared with the baseline findings. After five cycles of chemo-immunotherapy, this advanced patient still benefited and was changed to maintenance immunotherapy monotherapy. This case suggests that chemo-immunotherapy may provide an effective therapeutic option for those LCLC patients with low values of multiple predictive biomarkers, particularly for those who progressed from first-line classical treatments.

## Background

Primary lung tumors have been categorized into four major histological types: adenocarcinoma, squamous cell carcinoma, large cell carcinoma and small cell carcinoma (1, 2). Large cell lung carcinoma (LCLC) often occurs in the periphery of the lung rather than in the center and can be distinguished from the other lung cancers based on pathological findings (1, 2). To date, patients with LCLC are treated individually on the context of different histological phenotypes and gene mutations (3). For instance, gene alterations or rearrangements at EGFR, KRAS, TP53, ROS1 and ALK have already been identified in LCLC for appropriate selections of targeted therapy (3).

For those lung cancer patients without targetable driver mutations, platinum-based chemotherapy still served as standard treatments (4). Unfortunately, many patients may develop treatment-related resistance which can lead to disease progression. Recently, immune checkpoint inhibitors (ICIs) have sustained responses in different cancer types, particularly non-small cell lung cancers (NSCLC) (5). Thus, anti-cancer treatments of advanced NSCLC in patients without driver mutations have switched to chemo-immunotherapy from traditional chemotherapy alone. Furthermore, predictive biomarkers like PD-L1 expression or tumor mutational burden (TMB) has also been recommended for the prognosis of NSCLC (6). However, investigations on either the benefit role of ICIs or the clinical relevance of multiple predictive biomarkers in LCLC are very limited.

In this study, we presented a rare case of an advanced LCLC patient with low PD-L1 expression, low TMB value, and low density of CD8^+^ tumor-infiltrating lymphocytes (TILs), who still can effectively response to chemo-immunotherapy and the following maintenance immunotherapy monotherapy.

## Case Presentation

A 30-year-old non-smoker male with no medical or family history suffered from mostly unexplained cough with occasional bloody sputum and presented with a mass of the left upper lobe, multiple high-density round foci, and extensive lymphadenopathy of the lungs according to general computed tomography (CT) in January 2020. The serum tumor biomarkers of NSE (161.80 ng/ml) and CA125 (73.81 U/ml) were at high levels, while other biomarkers like CA153, CA199, and CEA were within normal range. Enhanced CT manifestations of the chest and abdomen on February 2020 revealed occlusion of the anterior segment of the left upper lung accompanied by soft tissue mass in periphery, tumor thrombus in left superior pulmonary artery and vein, lymph node metastasis in mediastinum, hilum, retroperitoneum, and left periosteum, multiple metastases of both lungs and left adrenal gland. Magnetic resonance imaging (MRI) showed no obvious abnormal body, bone, or brain metastasis. Then, fiberoptic bronchoscopy demonstrated diseased bilateral bronchus, and the corresponding pathological result showed poor differentiated large cell tumor with CK7 (+), CK-Pan (++), Ki-67 (+, 80%), CK5 (−), Naspin A (−), P40 (−), TIF (−), ERG (−), S100 (−), CgA (−), Syn (−), CD 56 (−). Additionally, a CT-guided percutaneous lung biopsy was carried out in the right lung, still showing poor differentiated large cell lung cancer with CK(L) (+), CK7(+), Ki-67 (+, 70%), Vinentin (−), P40 (−), NSE (−), NUT (−). Taken together, he was diagnosed as stage IV (cT4N3M1) LCLC.

Based on next-generation sequencing (NGS) with a small customized panel, no driver gene mutations of ALK, BRAF, EGFR, KRAS, MET, RET, ROS1 were observed. Therefore, this patient started first-line chemotherapy with pemetrexed (800 mg/kg, d1) plus lobaplatin (50 mg/kg, d1), and endosta (30 mg/day, 7 days) on March 1, 2020. After one-cycle chemotherapy, fiberoptic bronchoscopy demonstrated progressed disease with seriously blocked bronchus of the left lung and mucosal necrosis of anterior segment of the right upper lobe and the basal of the right lower lobe. Bronchial recanalization or resection of proliferative tissue was carried out in left bronchus. Meanwhile, CT scans on March 25, 2020 showed new metastases in the bilateral neck, as well as enlarged metastasis of the left adrenal gland and multiple lesions of the lungs. The serum levels of NSE and CA125 were increased to 274.90 ng/ml and 86.75 U/ml respectively, while other biomarkers were at normal levels.

Extensive NGS panel (YuceOne™ Plus X, Yucebio, China) results showed a somatic gene mutation found in *ATRX* Q929E, a TMB of 2.5 muts/mb, and a tumor neoantigen burden (TNB) of 0.5 neos/mb. By using a Dako PD-L1 22C3 pharmDx assay, immunohistochemistry staining showed a low PD-L1 expression with tumor proportion score (TPS) of 5–10%. Besides, additional immunohistochemistry findings illustrated a low CD8+ TIL density of 147.91 psc/mm² in this patient. Considering that combination chemotherapy and immunotherapy has been recommended as the most effective treatment in metastatic NSCLC patients with positive PD-L1 expression levels of 1–49%, he was then switched to pembrolizumab (200 mg mg/kg, d1) combining paclitaxel (400 mg mg/kg, d1) plus cisplatin (120 mg mg/kg, d2) on March 29, 2020. The lymph nodes in the bilateral neck lessened and pulmonary symptoms improved effectively after one cycle combination chemotherapy and immunotherapy on April 17, 2020. The serum concentrations of NSE and CA125 were decreased to 19.58 ng/ml and 54.75 U/ml respectively, while other biomarkers were at normal levels. With another cycle treatment, the lymph nodes in mediastinum, lung hilum, retroperitoneum and left periosteum obviously shrunk on CT scan on May 6, 2020 compared with the results on baseline CT imaging. In addition, fiberoptic bronchoscopy also showed improved disease, and other clinical symptoms were relieved. After further treatment with chemo-immunotherapy, partial response was maintained based on fiberoptic bronchoscopy and CT scans on June 19, 2020. The serum concentrations of NSE and CA125 returned to normal levels. Based on further follow-up, CT examinations after five cycles of chemo-immunotherapy on August 04, 2020 showed the lesion of the left lung seemed to partially progress, but other multiple lymph node metastases and the foci of the abdomen and pelvic still lessened. The serum concentrations of all serum biomarkers were within normal range. Then, he was changed to maintenance immunotherapy monotherapy with pembrolizumab (200 mg mg/kg, d1) on August 5, 2020. This patient maintained stable response and continued with maintenance pembrolizumab monotherapy till November 2020. Overall, this advanced LCLC patient benefited from chemo-immunotherapy and the following maintenance immunotherapy monotherapy when rapidly progressed after classical chemotherapy.

**Figure 1:**
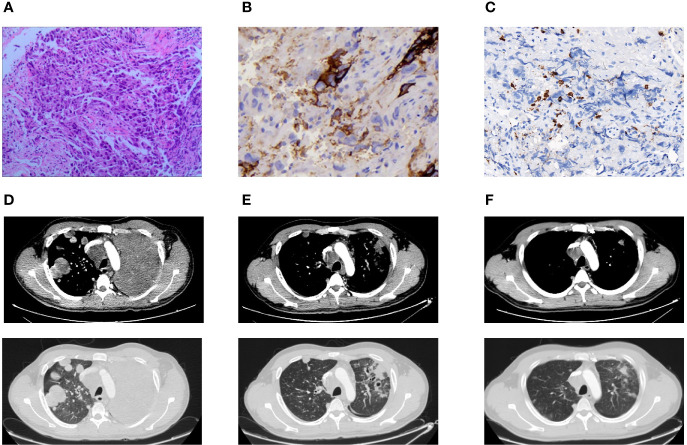
Pathologic findings and CT findings. **(A)** H&E stain demonstrated poorly differentiated carcinoma (×100). **(B)** Immunohistochemical stain showed a low positive PD-L1 expression of TPS 5–10% (×400). **(C)** Immunohistochemical stain indicated a low CD8(+) TIL density of 147.91 psc/mm² (×400). **(D)** Pulmonary lesions progressed in CT chest after one cycle of chemotherapy. **(E)** Pulmonary lesions lessened after two cycles of chemo-immunotherapy. **(F)** Pulmonary lesions shrunk after four cycles of chemo-immunotherapy.

**Figure 2:**
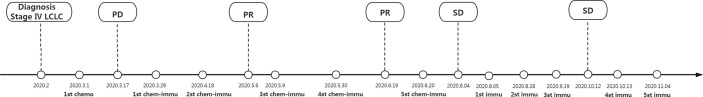
Time line and therapeutic regime. LCLC, large cell lung cancer; PR, partial response; PD, progressive disease; chemo, chemotherapy of pemetrexed (800 mg/kg, d1) plus lobaplatin (50 mg, d1) and endosta (30 mg/day, 7days); chemo-immu, chemo-immunotherapy of pembrolizumab (200 mg/kg, d1) combining paclitaxel (400 mg/kg, d1) plus cisplatin (120 mg/kg, d2); immu, immunotherapy monotherapy of pembrolizumab (200 mg/kg, d1). This patient was diagnosed as stage IV (cT4N3M1) LCLC for diseased lungs and multiple metastases or lymph node enlargements based on fiberoptic bronchoscopy and CT imaging, together with pathological findings showing poor differentiated large cell tumor. Due to no driver gene mutations, this patient was initiated with classical chemotherapy. After disease progression, he turned to chemo-immunotherapy regime for a positive PD-L1 expression (TPS 5–10%). Compared with the baseline findings of fiberoptic bronchoscopy and CT imaging, this advanced LCLC patient benefited from chemo-immunotherapy and the following maintenance immunotherapy monotherapy when rapidly progressed after classical chemotherapy till November 2020.

Pathologic findings and CT findings were shown in **Figure 1**, while time line and therapeutic regime were displayed in **Figure 2**. This patient has been tolerant without any untoward side effects during treatment or during follow-up, and he was considered for further lines of chemo-immunotherapy.

## Discussion

In recent years, PD-1 inhibitors (nivolumab and pembrolizumab) or PD-L1 inhibitors (atezolizumab, avelumab, and durvalumab) have revolutionized the treatments of lung cancers. Based on the results of clinical trial KEYNOTE 024 (*n = 305*), pembrolizumab has been approved by the FDA as the front-line therapy for advanced lung cancer patients with high PD-L1 expressions (TPS >50%) and without EGFR or ALK gene mutations (7). After that, the clinical study KEYNOTE 042 (*n = 1,274*) led to another FDA approval of pembrolizumab as the front-line monotherapy for stage III NSCLC patients who are inappropriate for surgery or definitive chemo-radiotherapy, or metastatic NSCLC (8). Also, tumors from these patients must have no EGFR or ALK gene aberration and be determined as positive PD-L1 expression (TPS ≥1%). But, patients with high PD-L1 expressions (TPS >50%) showed better clinical outcomes of pembrolizumab monotherapy than those with positive PD-L1 expression (TPS 1–49%) (8).

For NSCLC patients with a positive PD-L1 expression (TPS 1–49%), combination chemotherapy and immunotherapy has been recommended as the most effective treatment (4). The open-label phase II; study KEYNOTE 021 manifested that the objective response in metastatic non-squamous NSCLC patients has been greatly improved in the pembrolizumab plus chemotherapy group than in the chemotherapy alone group (*n = 123*, 55 *vs* 29%; *P* < 0.01) (9). Similar findings were found in another phase 3 clinical trial KEYNOTE 189, showing that combination strategy of pembrolizumab and pemetrexed-based chemotherapy resulted in extended progression-free survival in non-squamous NSCLC patients (*n = 616*, 8.8 *vs* 4.9 months; *P* < 0.01) (10) and overall survival in squamous NSCLC patients (*n = 559*, 15.9 *vs* 11.3 months; *P* < 0.01) (11). These clinical data of combination therapy with chemotherapy and pembrolizumab may provide accumulating evidence for the antitumor activity of chemotherapy not only involving direct cytotoxic effects but also relying on activation of tumor-targeting immune responses (12, 13).

Although PD-L1 expression may help clinicians to choose single-agent immunotherapy for high PD-L1 NSCLC (TPS >50%) or chemo-immunotherapy for positive PD-L1 NSCLC (TPS 1–49%), the variability in immunohistochemical staining antibodies and its heterogeneous expression may result in broad inconsistency of this biomarker (14–16). Thus, in clinical practice, some other predictive biomarkers emerged for immunotherapy. Taking advantages of whole-exome sequencing or specific gene panels, TMB has been evaluated as a potential predictive biomarker for anti-cancer immunotherapy (17, 18). TMB is typically calculated from the numbers of non-synonymous tumor-derived mutations dividing the sum in the genomic regions of the capture panel and can be used to estimate overall neoantigen load and to evaluate tumor immunogenicity. It is supposed that high TMB levels were associated with increased antigenic neoepitopes that can drive immunogenic host response and correlated with improved outcomes with immunotherapy (17, 18). Based on the data from the phase 2 clinical trial KEYNOTE 158, FDA approved the accelerated approval of pembrolizumab for adult and pediatric patients with unresectable or metastatic solid tumors determined as high TMB level (≥10 mut/Mb) (19). Likewise, this is also supported by a recent meta-analysis of 117 clinical trials, which reveals that high TMB (≥10 mut/Mb) was significantly correlated with enhanced objective response rates for ICIs (*P < 0.0001* for all) (20). Nevertheless, this FDA approval generated considerable arguments within the oncology community for lots of reasons (21). Notably, NSCLC patients were not recruited in KEYNOTE 158. Therefore, whether this FDA approval will impact the ICI therapies or chemo- or radio-immunotherapy for NSCLC is still unclear. To date, associations between high TMB and responses to PD-1/PD-L1 inhibitors have still been observed in many retrospective studies and real-world clinical practices, especially on cutoff values and tumor heterogeneity (22).

Due to the complex mechanisms for tumor immunity, in-depth molecular analyses with tumors and TILs in tumor microenvironments might also be important for underlying potential factors for the promotion of tumor immunogenicity (23). Accordingly, it is suggested to classify tumors into four different types according to the presence or absence of PD-L1 expression and CD8+ TILs. Immune type I patients present high PD-L1 expression and CD8+ TLs in the tumor microenvironment, and are responders to ICIs (24, 25). High density of CD8+ T cytotoxic cells was found to be associated with improved prognostic outcomes in patients with stage I–III NSCLC (26, 27). Additionally, some studies have also suggested that CD8+ TILs are correlated with PD-L1 expression in NSCLC patients treated with ICIs (28, 29). Recently, combination of CD8+ TILs, PD-L1 expression, and TMB has been exhibited to be associated with reliable prognosis in advanced NSCLC patients treating with ICIs (30).

Of late, it is reported that approximately 50% of LCLC patients lack a genetic profile of recognizable lineage-specific alterations, and 80% of LCLC patients are determined as a positive PD-L1 expression of TPS 1% (3). Besides, rare cases have been reported on pulmonary large cell neuroendocrine carcinoma treated with immunotherapy (31, 32), while even rare cases have been reported on the predictive role of single biomarker in LCLC (33). This might provide a potential clinical option of combination immunotherapy or immunotherapy alone in LCLC patients. To our best knowledge, this was the first case investigating the clinical relevance of multiple predictive biomarkers and therapeutic role of chemo-immunotherapy in LCLC. This present case signified a potential advantage of chemo-immunotherapy in advanced LCLC patients with no driver mutations, even if they had low TMB, PD-L1 expression and low CD8^+^ TIL density. Obviously, there were a large number of patients with low levels of TMB, PD-L1 expression, and TIL density not only in LCLC, but also in other lung cancers or other types of cancer. Thus, this case suggests that chemo-immunotherapy may provide a potential effective therapeutic option for the managements of these patients.

In conclusion, chemo-immunotherapy provides a potential therapeutic option for LCLC patients with low values of multiple predictive biomarkers, particularly for those who progressed from first-line classical treatment. Further studies will be performed to better clarify the therapeutic efficacy and potential role of chemo-immunotherapy in LCLC.

## Data Availability Statement

The raw data supporting the conclusions of this article will be made available by the authors, without undue reservation.

## Ethics Statement

Written informed consent was obtained from the individual(s) for the publication of any potentially identifiable images or data included in this article.

## Author Contributions

GW and QC conceptualized and designed the study. YX interpreted the data and prepared the manuscript. WP, MT, and JW, followed-up the patient and collected the data. HL and XS revised and edited the manuscript. LW was the principle investigator. All authors contributed to the article and approved the submitted version.

## Funding

This study was kindly funded by Hunan Cancer Hospital Climb Plan (NO. ZX2020005-5) and Hunan Province Health Commission Foundation (NO.B2019090).

## Conflict of Interest

YX, MT, HL and XS were employed by the company YuceBio Technology Co., Ltd.

The remaining authors declare that the research was conducted in the absence of any commercial or financial relationships that could be construed as a potential conflict of interest.
